# Irrational prescribing of over-the-counter (OTC) medicines in general practice: testing the feasibility of an educational intervention among physicians in five European countries

**DOI:** 10.1186/1471-2296-15-34

**Published:** 2014-02-17

**Authors:** Christos Lionis, Elena Petelos, Sue Shea, Georgia Bagiartaki, Ioanna G Tsiligianni, Apostolos Kamekis, Vasiliki Tsiantou, Maria Papadakaki, Athina Tatsioni, Joanna Moschandreas, Aristoula Saridaki, Antonios Bertsias, Tomas Faresjö, Åshild Faresjö, Luc Martinez, Dominic Agius, Yesim Uncu, George Samoutis, Jiri Vlcek, Abobakr Abasaeed, Bodossakis Merkouris

**Affiliations:** 1 Clinic of Social and Family Medicine, Faculty of Medicine, University of Crete, Voutes, PO BOX 2208, Heraklion, P.C. 71003, Greece; 2 Department of Health Economics, National School of Public Health, Alexandras Avenue 196, Athens 11521, Greece; 3 Department of Internal Medicine, University of Ioannina School of Medicine, Ioannina 45110, Greece; 4 Biostatistics Lab, Faculty of Medicine, University of Crete, Voutes, PO BOX 2208, Heraklion P.C. 71003, Greece; 5 Department of Medicine and Health/Community Medicine, Faculty of Health Sciences, Linköping University, Linköping SE-581 83, Sweden; 6 Department of general Practice, UMR_S 136, Sorbonne University, UPMC Univ Paris 06, Paris, France; 7 Mediterranean Institute of Primary Care, Attard ATD 1300, Malta; 8 Turkish Association of Family Physicians (TAHUD), 79. Sokak, No:4/5, Emek, 06510 Ankara, Turkey; 9 University of Nicosia Medical School, Messinis 3, 2301 Nicosia, Cyprus; 10 Faculty of Pharmacy in Hradec Kralove, Charles University in Prague, Prague, Czech Republick; 11 Greek Association of General Practitioners, Kountouriotou 21, Thessaloniki 54625, Greece; 12 Pierre Louis Epidemiology and Public Health Institute, EPAR Team, F-75013 Paris, France

**Keywords:** OTC medicines, Primary care, Feasibility study

## Abstract

**Background:**

Irrational prescribing of over-the-counter (OTC) medicines in general practice is common in Southern Europe. Recent findings from a research project funded by the European Commission (FP7), the “OTC SOCIOMED”, conducted in seven European countries, indicate that physicians in countries in the Mediterranean Europe region prescribe medicines to a higher degree in comparison to physicians in other participating European countries. In light of these findings, a feasibility study has been designed to explore the acceptance of a pilot educational intervention targeting physicians in general practice in various settings in the Mediterranean Europe region.

**Methods:**

This feasibility study utilized an educational intervention was designed using the Theory of Planned Behaviour (TPB). It took place in geographically-defined primary care areas in Cyprus, France, Greece, Malta, and Turkey. General Practitioners (GPs) were recruited in each country and randomly assigned into two study groups in each of the participating countries. The intervention included a one-day intensive training programme, a poster presentation, and regular visits of trained professionals to the workplaces of participants. Reminder messages and email messages were, also, sent to participants over a 4-week period. A pre- and post-test evaluation study design with quantitative and qualitative data was employed. The primary outcome of this feasibility pilot intervention was to reduce GPs’ intention to provide medicines following the educational intervention, and its secondary outcomes included a reduction of prescribed medicines following the intervention, as well as an assessment of its practicality and acceptance by the participating GPs.

**Results:**

Median intention scores in the intervention groups were reduced, following the educational intervention, in comparison to the control group. Descriptive analysis of related questions indicated a high overall acceptance and perceived practicality of the intervention programme by GPs, with median scores above 5 on a 7-point Likert scale.

**Conclusions:**

Evidence from this intervention will estimate the parameters required to design a larger study aimed at assessing the effectiveness of such educational interventions. In addition, it could also help inform health policy makers and decision makers regarding the management of behavioural changes in the prescribing patterns of physicians in Mediterranean Europe, particularly in Southern European countries.

## Background

Patient safety has been noted as an important area of public health care and an increasingly growing area of health services and policy research over the past few years [1]. The focus of such research tends to be mainly on hospital care, [2] whereas major sources of harm within primary care settings have yet to be fully explored [3]. There is evidence that irrational prescribing in primary care may introduce considerable harm, resulting in a number of hospital admissions due to adverse drug events [4]. Furthermore, a recent study indicated high-risk prescribing was more common in primary care patients who were being prescribed medicines intended for long-term use [3]*.* Based on the rational prescribing definition of the World Health Organisation (http://www.who.int/mediacentre/factsheets/fs338/en/), rational use of medicines requires that “*patients receive medications appropriate to their clinical needs, in doses that meet their own individual requirements, for an adequate period of time, and at the lowest cost to them and their community*”.

However, trends in recent decades include the changing status in the provision of medicines from Prescription-Only Medicines (POMs) to Over-The-Counter (OTC) medicines, frequently provided without prescription for minor ailments in many countries. Although the use of OTC medicines is steadily rising, [5] and concerns about inappropriate treatment and adverse medicine reactions have been raised, [6] this subject does not appear to have received the attention it deserves in general practice research, resulting in a serious evidence gap, particularly in Europe [7,8].

Problems relating to irrational prescribing, provision and use of both POMs and OTC medicines, and the subsequent impact on patient safety, appear to be more severe in countries without a well-organized primary care system or in countries where a gap exists between legislation and practice. In Greece, for example, although many medicines are not specifically defined by the existing legislation as OTCs, they can still be obtained without prescription. Antibiotics are such an example, with Greece being ranked as having one of the highest antimicrobial resistance rates in Europe [9]. This problem is further exacerbated as Greek primary care patients have a tendency to often exchange OTC medicines with friends and relatives without seeking advice from either their General Practitioner (GP) [7] or pharmacist.

There is a current discussion about the role of GPs regarding their role in monitoring the use of the OTC medicines by their patients, and there is evidence that well-trained GPs can reduce the irrational use of OTC medicines and, thus, improve patient safety [10]. Within this context, a European project was developed, receiving funding by the European Commission through the Seventh Framework Programme (FP7), and focusing on “*Assessing The Over-The-Counter Medications In Primary Care And Translating The Theory Of Planned Behaviour Into Interventions (OTC SOCIOMED)”(EU 7th FP n°223654-06/05/08)”.* Its primary objective was to assess the extent of irrational prescribing jointly with the provision of OTC medicines in Southern European countries and to identify factors which influence the intention of GPs and pharmacists towards the provision of OTC medicines and the intention of patients/clients towards the consumption of OTC medicines. The design and implementation of a pilot intervention has been included among the objectives of this European collaborative project. Within the framework of this project, it was deemed important to explore the extent to which the empirical and descriptive research implemented in this European project could be translated into actions and policy. Educational interventions have been shown to improve the quality of prescribing, which in turn may lead to reduction in polypharmacy and its associated high societal costs [11]. GPs and other frontline physicians serving in primary care appear to be an appropriate group of health care professionals to target in such interventions [12]. Although early conceptual models of health education and modern versions of health promotion indicate that interventions should focus on changeable behaviours and objectives [13], interventions testing behavioural models in this area of medical research are scarce.

From the empirical and descriptive research that was carried out in the first steps of this project, we identified that positive attitude towards prescribed medicines and social pressure (subjective norm category of the theory of planed behaviour) was found to affect the GPs’ intention to provide medicines. In addition, a gender difference was found, with women GPs appearing to be more likely to provide a greater number of medicines to their patients compared to their male colleagues. Based on these observational findings we designed a pilot educational intervention with the aim of establishing the extent to which the GPs’ intention and behaviour in terms of medicine provision, mostly on prescribing and advice on the use of OTC medicines, may be affected by the intervention. We purposively focused on GPs only and not on other individuals (i.e. patients and pharmacists) regarding medicine provision and use. The reason for this was because we wanted to measure the effect of the intervention on GPs without the measured effect being artificially enhanced by the involvement of other partners, and we wished to draw conclusions specifically on the acceptance of such an intervention by this group. Our key research question and interest was to explore whether the implemented educational intervention in primary care would be effective, feasible and acceptable to be utilized prospectively as the basis of a larger scale study.

Thus, the main aim of this paper is to report on the design of this educational intervention, as well as the measured outcomes of its implementation. Among the objectives of this study was the presentation of the key components of the study with a focus on the psychological constructs to predict clinical behaviour and explore the acceptance and practicality of this educational intervention.

### Theoretical framework

The TPB was used as the theoretical framework for the design of this feasibility study. The TPB seeks to explain why people perform certain actions. According to the TPB, a person’s intentions are a good predictor of their behaviour. The stronger the intention to perform a particular behaviour, the more likely the person is to perform that behaviour. The model states that the intention to carry out an action is influenced by the person’s beliefs (behavioural attitudes), the social pressure to conform to the expectations of others (subjective norms), and their perceived ability to carry out the action (perceived behavioural control) [14].

TPB states that attitudes towards behaviour are determined by the individual’s evaluation of the outcomes associated with the behaviour. The more positively the person evaluates the likely outcomes and believes that the behaviour will achieve these outcomes, the more likely it is that this person will perform the behaviour. Subjective norms refer to the extent to which a person believes that significant individuals or groups (e.g. parents, spouse, close friend, co-workers, doctor or accountant) will approve or disapprove of their performing the behaviour. The more the person believes that people with whom he or she is motivated to comply think that he or she should perform the behaviour, the more likely it is that the person will feel social pressure to perform this behaviour [15]. The TPB has been known to be a useful method to identify factors relevant to prescribing patterns of GPs within this same European research project [16].

Finally, perceived behavioural control refers to the extent to which the individual believes they can control their behaviour and this includes beliefs about factors that may hinder or promote the behaviour. The more a person believes s/he has control over the action to be performed, the more likely s/he is to perform the particular behaviour [15].

## Methods

### Design

A feasibility study was designed to assist the OTC SOCIOMED FP7 project and in particular to assess the acceptance and practicality of the implemented pilot educational intervention study. The project received approval by local authorities and National Bioethics Committees in the participating countries (CY No: EEBK EP2010 01.16; FR No: EGY/NDS/AR105323; GR No: 4483/31-5-2010; MT No: HEC23/10-07.10.2010; TR No: 2010-6/1).

### Study setting

One to two geographically-defined Primary Health Care (PHC) areas in each of the five Mediterranean countries, (Cyprus, France, Greece, Malta and Turkey), were selected for the pilot intervention, representing a mix of urban, rural and semi-urban distinct setting characteristics within the GP community. For clarification purposes and for reasons serving the research questions, the participating countries were divided into Eastern Mediterranean countries (Cyprus, Greece, Malta and Turkey) and Western Mediterranean countries (France). The primary care system that is currently operating in the countries of the East Mediterranean basin, including the countries of Cyprus, Greece, Turkey and Malta presents many similarities, particularly at the time that the study was carried out. Primary health care centres that deliver their services on a 24-hour basis, jointly with their peripheral posts, seem to be the predominant model in rural areas in Malta, Greece, and Cyprus; whilst this is a fact based on empirical evidence, the number of GPs who work in solo practice and serve the private sector appears to be increasing in these countries. The specialty of general/family practice has been recognized in all participating settings and only certified GPs were invited to participate in our study.

Geographically-defined Primary Health Care areas were established on the basis of the characteristics of the regional organization for each primary health care system; these definitions were extensively discussed and clarified during the first meeting of the OTC SOCIOMED executive board to avoid variation of definition in terms of these characteristics across countries. All GP practices, public (health centres and satellite practices) or private (solo and group practices), in these areas were eligible for participation in the feasibility study (CY = 28, FR = 9200 (only solo practices), GR = 66, MT = 72, TR = 21). A number of GP practices in each study setting were then selected out of the total eligible GP practices to be involved in the study, based on the GPs’ acceptance to participate in the study, with the exception of Malta, where all the eligible GP practices were included in the study (CY = 10, FR = 527, GR = 18, MT = 72, TR = 10). All the practising GPs serving the selected GP practices in each study setting were invited to participate in the study (CY = 76, FR = 527, GR = 34, MT = 90, TR = 41). The GPs who accepted this invitation were the participants of this feasibility study (CY = 10, FR = 9, GR = 17, MT = 25, TR = 23). Informed consent was obtained from all the study participants prior to participation. Allocation of the GPs to the study groups was made randomly in most settings, through assigning a unique code to each GP, with the exception of Cyprus, where allocation was based on the GPs’ availability to engage in the intervention. Allocation of GPs to the study groups in all the study settings was based on the GP practice they served in order to avoid the risk of contamination of individual GPs from information diffusion. For these reasons, all participating GPs in any given practice were allocated either to the intervention group or to the control group.

### Study description and implementation

The feasibility study was implemented in two phases, a preparatory stage, where the observational findings from this project were utilised for the design of the pilot educational intervention study (Phase 1), and a second phase including both the implementation and evaluation of this intervention (Phase 2).

#### Phase 1: Translating the findings of the OTC SOCIOMED project into an intervention design

The key findings of a survey conducted in seven countries in the framework of the OTC SOCIOMED project (work packages 3 and 4, http://www.otcsociomed.uoc.gr), and employing the TPB model to assess factors influencing the beliefs and attitudes of GPs, pharmacists and patients/clients OTC medicines guided the design, content and methods of this intervention study. For the purpose of this European project we used the phrase “provision of medicines” for both prescribing and recommending of medicines, based on local laws and regulations regarding supply of medicines.

##### Translating evidence from international literature into an intervention design

A systematic review of international literature was also conducted within the OTC SOCIOMED project to better inform the design of this feasibility study, given the fact that implementation of interventions in the area of OTCs is still a neglected subject in general practice/family medicine. Findings of this review are presented in a separate paper, but we have opted to mention those elements from our findings deemed to be the most relevant for the purposes of the OTC SOCIOMED project. The results of this review suggest evidence is lacking for this particular topic, but that common components of intervention studies within the last 10 years include educational material and practice guidelines [17-22], computerised information and timetables [23], conferences, seminars, workshops or lectures [24-33] educational outreach visits [33-35], patient and computerised feedback [36], reminders [37], graphical displays [38], use of opinion of patients and advisors-educators [39-41] and mailing questionnaires [42]. Evidence from previous systematic reviews included in the scoping of this project supports high effectiveness of structural [43] and multifaceted interventions focusing on multiple targets, compared to single and individual interventions [44].

Additionally, there were no studies focusing on comparative intervention effectiveness or studies “borrowing” from the social sciences in terms of behavioural theories and attempting to design an intervention in an interdisciplinary fashion. The review revealed limited evidence in terms of the effectiveness of the interventions on prescribed medicines in general practice and a serious evidence gap on OTC rational provision and consumption. In terms of the effectiveness of the interventions assessed, educational types of intervention (e.g. courses, aids, campaigns, face-to-face, academic detailing) appeared to be the most promising in improving prescribing behaviour [24,45].

The aforementioned interventions were studied in depth to help us structure our own intervention. Nevertheless, it is important to mention in some cases the observable effect was either not sustained for long periods of time or not reported upon. In certain cases, even though the intervention may have worked, the quality was assessed as a quantifiable outcome and/or there were no specifically-selected quality indicators to assess, for example, the long-term effectiveness and sustainability of the intervention, as the majority of the goals in the interventions were simply to reduce the number of prescribed medicines [46] and assess this at a specific point in the near future following the implementation of the intervention.

#### Phase 2: Implementation and evaluation of the intervention

The intervention was designed on the basis of the TPB, which has been identified as a promising model for behaviour change in general practice [47]. The content of the intervention was culturally specific and developed according to local legislation and requirements regarding prescribing for the various participating countries. It included three main components: (a) the delivery of a one-day educational course (b) poster demonstration with key messages on medicine prescribing over a 4-week period, and, (c) regular visits by a trained health care professional (acting as informant) to the workplace of the participants, coupled with reminder messages and email messages over the 4-week period of the intervention.

All GPs allocated to the intervention group in each participating country were invited to attend an intensive one-day educational course. Issues on how GPs should educate their patients regarding the risks of irrational use of OTC medicines were addressed through the training, together with promoting collaboration with pharmacists. The course employed various educational techniques such as lectures, role-play and small group discussions. Key lectures informed the participants about the available literature on the principles of rational prescribing, adverse reactions to drugs, drug interactions and health risks related to their misuse [24,48].

The educational course, also, included interactive training methods in addition to conventional lectures, while a number of clinical scenarios were used to stimulate debate on the treatment options in the small group discussions [27,49]. Role-play procedures were also employed to encourage participants to become actively involved in the discussion.

Supporting materials, such as posters placed at the workplace of participants and alert messages (text or email messages) for physicians, were used during the intervention as “reminder tools” aimed at raising participant awareness [50]. The reminder tools contained short and concise messages related to the prevention of irrational use of medicines, the careful provision of OTC medicines as well as the promotion of patient safety. Furthermore, regular visits by health care professionals were made to the workplace of the participants over a 4-week period; these health care professionals had previously received a 3-hour training session by the research team in each participating country. These visits involved personal communication with the study participants and delivery of written material, which served as reminders of the main aim and objectives of the intervention in the context of everyday practice.

### Instruments, measurements and outcomes of the pilot educational intervention study

#### 1) Training assessment questionnaire

The Training Assessment Questionnaire aimed to evaluate various aspects of the one-day training seminar. Seven-point Likert scale items related to the quality of the seminar and its speakers (1 = high/7 = low), its potential impact on GPs’ behaviour regarding OTC medicines and their practice regarding the issue of prescribing (1 = strongly disagree/7 = strongly agree) as well as its applicability on their future work (1 = not important/7 = important) were used. Questions in the Training Assessment Questionnaire were not analysed for France, since a different version of the questionnaire was employed.

#### 2) Complementary questionnaire on OTC medicines

A complementary questionnaire was used to elicit information focusing on OTC medicines. This 11-item questionnaire explored the attitudes and behaviour of GPs towards OTC medicines, and consultation about OTC medicines to their patients, and included closed and open-ended questions, as well as a series of scenarios based on real patient situations. These scenarios were developed on the basis of the key findings of this European project (Work packages 3 and 4) in the participating countries. The questionnaire was distributed to participating GPs serving both in the intervention and the control groups. It also contained post-intervention questions responded to by the participants in the intervention groups on a 7-point Likert scale (1 = very bad/7 = very good) in an attempt to collect quantitative and qualitative data regarding the intervention. The groups in France did not complete the post-intervention questionnaires due to local and organisational barriers.

#### 3) TPB questionnaire

This questionnaire was constructed under the guidance of a previously implemented FP5 project, the Research-Based Education and Quality Improvement (ReBEQI) project which aimed to create a framework for selecting and ensuring the implementation of interventions towards the improvement of quality of healthcare. The questionnaire was distributed to all participating GPs in both intervention and control groups [48]. It was initially developed and tested in the Greek language and was then translated into six European languages [16]. All the questionnaires utilised in this pilot study have been culturally tested prior to their implementation in the participating settings. A short version of the original questionnaire was administered before and after the intervention (pre- and post-intervention phases) with the aim to assess the variations in attitudes, social norms, perceived behaviour control and intentions regarding the provision and consumption of medicines. The primary focus was placed on three different dimensions of intention towards provision of medicines, namely “Generalized Intention towards medicine provision” (GI), “Intention Performance Statement 1” (IPS1), which expressed GP expectation to provide medicines, and “Intention Performance Statement 2” (IPS2), which expressed GP expectation to issue a prescription without having well-documented evidence about their patient. All items were measured on a seven-point Likert scale except for the Intention performance statements (IPS1 & IPS2), which were measured on a 10-point scale.

#### 4) Patient medication form

Provision of medicines was measured through the review and analysis of the medical records of five patients per GP in both groups (intervention and control) before and after the intervention. The first five consecutive patients aged 60 years and over, visiting the GPs’ practice to seek a prescription, were selected to participate in the study. The number of prescribed medicines was recorded before and after the intervention in order to identify changes in the provision of medicines [18,27]. The measure of interest was the difference between the number of medicines prescribed in the last visit before the intervention and the first visit after the intervention.

### Outcomes of the pilot educational and intervention study

The primary outcome of this study was to investigate if there was a reduction in GPs’ intention to prescribe medicines following the educational intervention. Intention to provide medicines was measured at pre- and post-intervention phases in both control and intervention groups by utilizing three TPB scales, namely GI, IPS1 and IPS2. A secondary outcome was to investigate whether there was an overall reduction in the number of medicines prescribed by GPs to a selected group of their patients following the intervention, and in comparison to the baseline. Secondary outcomes included the acceptance and practicality of the educational intervention as evaluated by the participating GPs.

#### Acceptance

The acceptance of this intervention was assessed by examining responses to questions regarding the organization and the content of the overall training. Additionally, the one-day seminar in its entirety, the quality of the speaker presentations and the overall quality of the intervention according to the expectations GPs had prior to attending the seminar were also evaluated. Acceptance-related questions were included in the Training Assessment Questionnaire as well as in the Complementary Questionnaire on OTC medicines. Overall, participating GPs were willing to be randomized into the two study groups; no problems were reported in the patient recruiting process. Internal consistency of Acceptance-related questions found in both questionnaires was satisfactory with Cronbach’s alpha index being a = 0.821 (in Complementary questionnaire) and a = 0.656 in Practicality-related items found in the Complementary Questionnaire and in 1-day training questionnaire respectively.

#### Practicality

The practicality of the intervention was evaluated via analyses of questions related with GPs’ work and practice. The aim was to assess whether GPs believed that this intervention programme could affect their practices in the matter of prescribing. Thus, GPs were invited to assess whether this intervention changed their view of OTC medicines, whether it was helpful for their future work, if the themes of the seminar changed their views in the issue of prescribing and their behaviour towards the use of non-prescribed drugs. These items were selected from both the Complementary Questionnaire on OTC medicines and the Training Assessment Questionnaire The internal consistency of practicality-related questions relevant for the 1-day training questionnaire was satisfactory (Cronbach’s alpha index a = 0.690).

### Data collection

Interviewers were employed in each participating country in order to collect and extract the data from all questionnaires. Electronic database files were filled in by each participating country by the participating GPs and subsequently sent for merging, management and analyses at the Biostatistics Laboratory, Faculty of Medicine of University of Crete, Greece, where a common database has been created.

### Data analysis

The acceptance and practicality of the intervention were evaluated using descriptive analysis of the items mentioned in the Study evaluation section. The Chi-square test of independence and the non-parametric Kruskal-Wallis test were applied in order to investigate differences in the sociodemographic characteristics of respondents. The Mann–Whitney non-parametric test was applied to compare differences between intervention and control group. The level of statistical significance was chosen to be 5% and the statistical software package used was IBM SPSS 19.

## Results

### Participant demographics

Eighty-four general practitioners participated in the study (Cyprus n = 10, France n = 9, Greece n = 17, Malta n = 25 and Turkey n = 23). The control group consisted of 48 GPs (Cyprus n = 5, France n = 5, Greece n = 12, Malta n = 14 and Turkey n = 12) and the intervention group of 36 GPs (Cyprus n = 5, France n = 4, Greece n = 5, Malta n = 11 and Turkey n = 11). The proportion of complete data was fairly high in the East Mediterranean countries (100% in Malta, Turkey and Cyprus, and 90.5% in Greece) yet lower in France where 45% of respondents completed all the study pre-intervention questionnaires including the follow-ups without missing data.

The sociodemographic characteristics of the GPs (Table 1) varied significantly amongst participants. Cypriot, Turkish and Greek physicians were on average younger (median 40 years in Cyprus and Turkey, and 42 years in Greece) than their Maltese (47 years) and French colleagues (52 years). Age variations matched the variation in the clinical experience of GPs; all French physicians had > 10 years of experience, whereas 70% of the Cypriot physicians had < 10 years of practice (p < 0.0001). The primary care setting also varied significantly among the participating countries, with Greek and Turkish physicians serving the public sector in Health Centres/Hospitals and the majority of French and Maltese physicians serving the private sector.

**Table 1:** Sociodemographic characteristics of respondents

	**Cuprus**	**France**	**Greece**	**Malta**	**Turkey**	**P-value**
**Total cases**	10	9	17	25	23	
**Gender**						0.703^1^
Male n (%)	8(80.0)	6(66.7)	9(52.9)	15(60.0)	15(62.5)	
Female n (%)	2(20.2)	3(33.3)	8(47.1)	10(40.0)	8(34.8)	
**Mean age**	40	52	42	47	40	0.002^2^
**Type of service area**						
Rural/Semi-urban/Other	4(40)	0(0)	17(100)	14(56.0)	17(73.9)	<0.0001^3^
Urban	6(60.0)	9(100)	0(0)	11(44.0)	6(26.1)	
**Years of experience**						
0-10 Years	7(70.0)	0(0)	11(64.7)	8(32.0)	3(13.0)	<0.0001^4^
>10Years	3(30.0)	9 (100)	6(35.3)	17(68.0)	20(87.0)	
**Kind of organization**						
Private/Combination/Don’t know/Other	4(40.0)	8(88.9)	0(0.0)	19(76.0)	0(0)	<0.0001^5^
Public	6(60)	1(11.1)	17(100)	6(24.0)	23(100)	
**Organizational type**						
Health center/Hospital	6(60.0)	0(0)	17(100)	8(32.0)	23(100)	<0.0001^6^
Independent/Chain/Group/Other	4(40.0)	9(100)	0(0)	17(68.0)	0(0)	

^1^The chi-squared test of independence was applied X^2^ = 6.499 (d.f. = 4).

^2^The non parametric Kruskal-Wallis Test was applied X^2^ = 18.132 (d.f. = 4).

^3^The chi-squared test of independence was applied X^2^ = 35.131 (d.f. = 4).

^4^The chi-squared test of independence was applied X^2^ = 26.502 (d.f. = 4).

^5^The chi-squared test of independence was applied X^2^ = 53.413 (d.f. = 4).

^6^The chi-squared test of independence was applied X^2^ = 53.519 (d.f. = 4).

### Intentions & number of medicines

Median scores and median differences in TPB measures concerning intentions are presented in Table 2. A pattern of positive changes was observed in the intervention groups in East Mediterranean countries, with the median intention scores being reduced after the intervention, whilst, smaller or no changes were observed in the respective control groups. Statistically significant differences were observed in Cyprus (GI; p-value = 0.018, IPS2; p-value = 0.017) and in Malta (IPS1; p-Value = 0.021). Besides TPB measures, prescription patterns were evaluated using the Patient Medication Form questionnaire. Information from this questionnaire is also presented in Table 2. There was no observable change in the number of prescribed medicines recorded (pre- and post-intervention) in Cyprus and Greece, while in France there was a small increase in the number of prescribed medication. In Malta and in Turkey a small decrease in the number of prescribed medicines was observed in both groups without it being statistically significant.

**Table 2:** GPs TPB intentions per country and group

	**Control group**		**Intervention group**		
**Country/Measure**	**Baseline median (Min, Max)**	Difference*: median (Min, Max)	**Baseline median (Min, Max)**	Difference*: median (Min, Max)	**P-Value**
**Cyprus**					
GI	4.0	0.0	6.5	1.0	0.018
	(3.0, 5.0)	(-1.0, 0.0)	(4.0, 7.0)	(0.0, 2.0)	
IPS1	8.0	0.0	8.00	1.00	0.419
	(6.0, 9.0)	(-1.0, 1.0)	(5.0, 10.0)	(0.00, 1.00)	
IPS2	0.0	0.00	2.0	2.0	0.017
	(0.0, 2.0)	(-1.0, 0.0)	(2.0, 7.0)	(0.0, 3.0)	
No. of prescribed medicines	4 (2, 7)	0 (0, 0)	4 (2, 4)	0 (0, 0)	1.000
**France**					
GI	4.5	0.0	4.5	0.5	0.707
	(3.0, 6.0)	(-3.0, 1.5)	(3.0, 6.0)	(-1.0, 4.0)	
IPS1	7.0	1.00	6.0	-0.5	0.209
	(6.0, 9.0)	(-1.0, 2.0)	(6.0, 7.0)	(-3.0, 1.0)	
IPS2	5.0	1.0	3.5	0.5	0.156
	(3.0, 7.0)	(0.0, 2.0)	(2.0, 5.0)	(-3.0, 1.0)	
No. of prescribed medicines	4 (2, 9)	-1 (-1, 2)	4 (2, 9)	-1 (-4, 2)	0.707
**Greece**					
GI	3.5	0.0	4.0	1.0	0.351
	(1.0, 6.0)	(-3.0, 3.5)	(2.5, 5.0)	(-1.5, 3.5)	
IPS1	5.5	0.0	6.0	1.0	0.340
	(2.0, 9.0)	(-3.0, 4.0)	(3.0, 8.0)	(0.0, 2.0)	
IPS2	3.0	0.0	4.0	1.0	0.227
	(0.0, 7.0)	(-2.0, 2.0)	(1.0, 8.0)	(-1.0, 4.0)	
No. of prescribed medicines	4	0	3	0	0.477
	(1, 6)	(-1, 1)	(1, 5)	(0, 0)	
**Malta**					
GI	4.0	0.5	4.3	1.0	0.108
	(2.0, 7.0)	(-1.5, 1.5)	(2.0, 7.0)	(-1.00, 3.0)	
IPS1	6.0	0.5	7.00	1.0	0.021
	(3.0, 9.0)	(-1.0, 3.0)	(4.0, 9.0)	(1.0, 4.0)	
IPS2	2.0	0.0	3.0	1.0	0.154
	(0.0, 9.0)	(-1.0, 2.0)	(0.0, 10.0)	(-1.0, 6.0)	
No. of prescribed medicines	3	1	3	1	0.152
	(2, 4)	(-1, 1)	(2, 5)	(0, 3)	
**Turkey**					
GI	4.5	0.8	4.0	0.0	0.419
	(2.50, 6.0)	(-3.0, 2.5)	(1.0, 6.0)	(-4.5, 2.0)	
IPS1	7.5	0.0	8.0	1.0	0.549
	(6.0, 10.0)	(-2.0 7.0)	(0.0, 9.0)	(-6.0, 4.0)	
IPS2	4.5	0.5	3.0	1.0	0.804
	(2.0, 9.0)	(-8.0, 8.0)	(0.0, 8.0)	(-5.0, 7.0)	
No. of prescribed medicines	4	1	4	1	0.612
	(2, 6)	(-1, 4)	(3, 7)	(0, 2)	

*Differences were computed for each measure by subtracting the scores after intervention from the baseline scores.

### Acceptance

**Figure 1:**
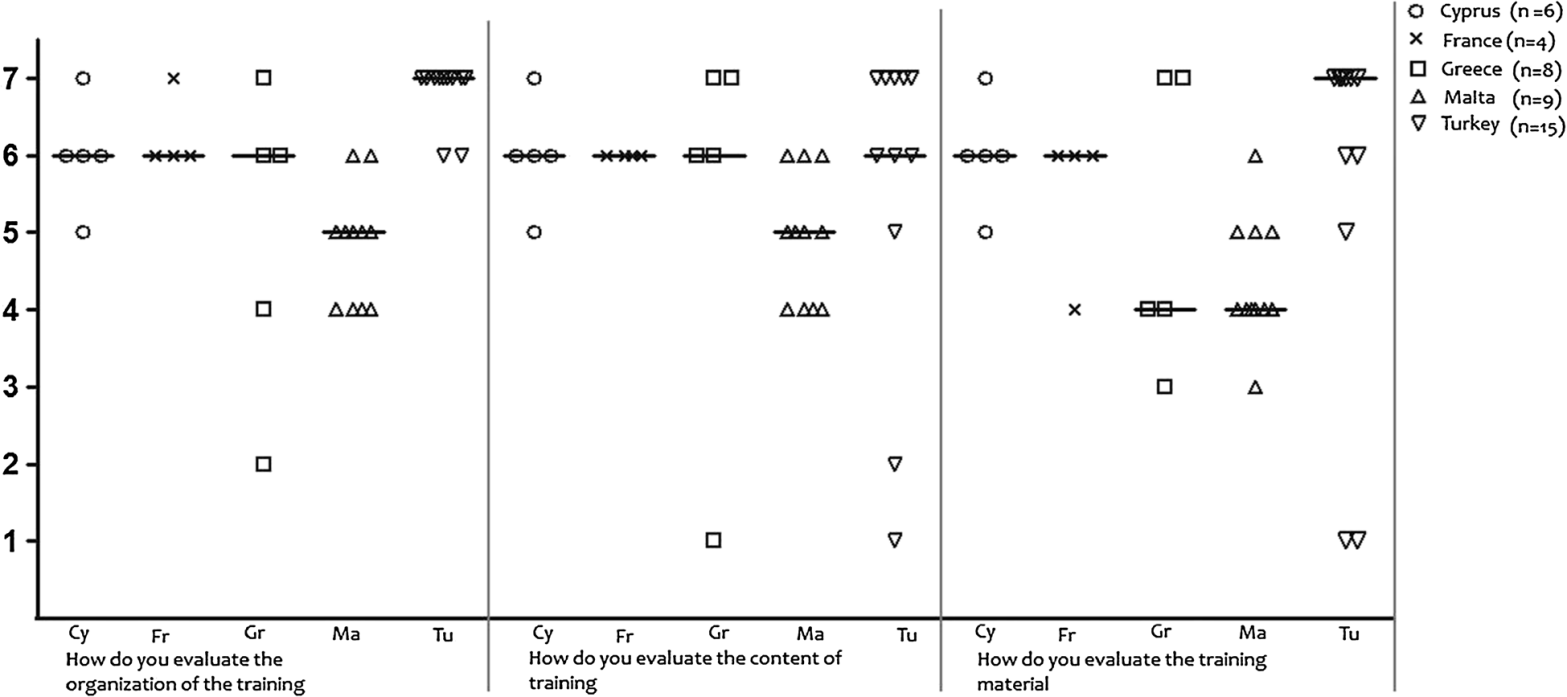
Acceptance related questions (complementary questionnaire on OTC medicines).

**Figure 2:**
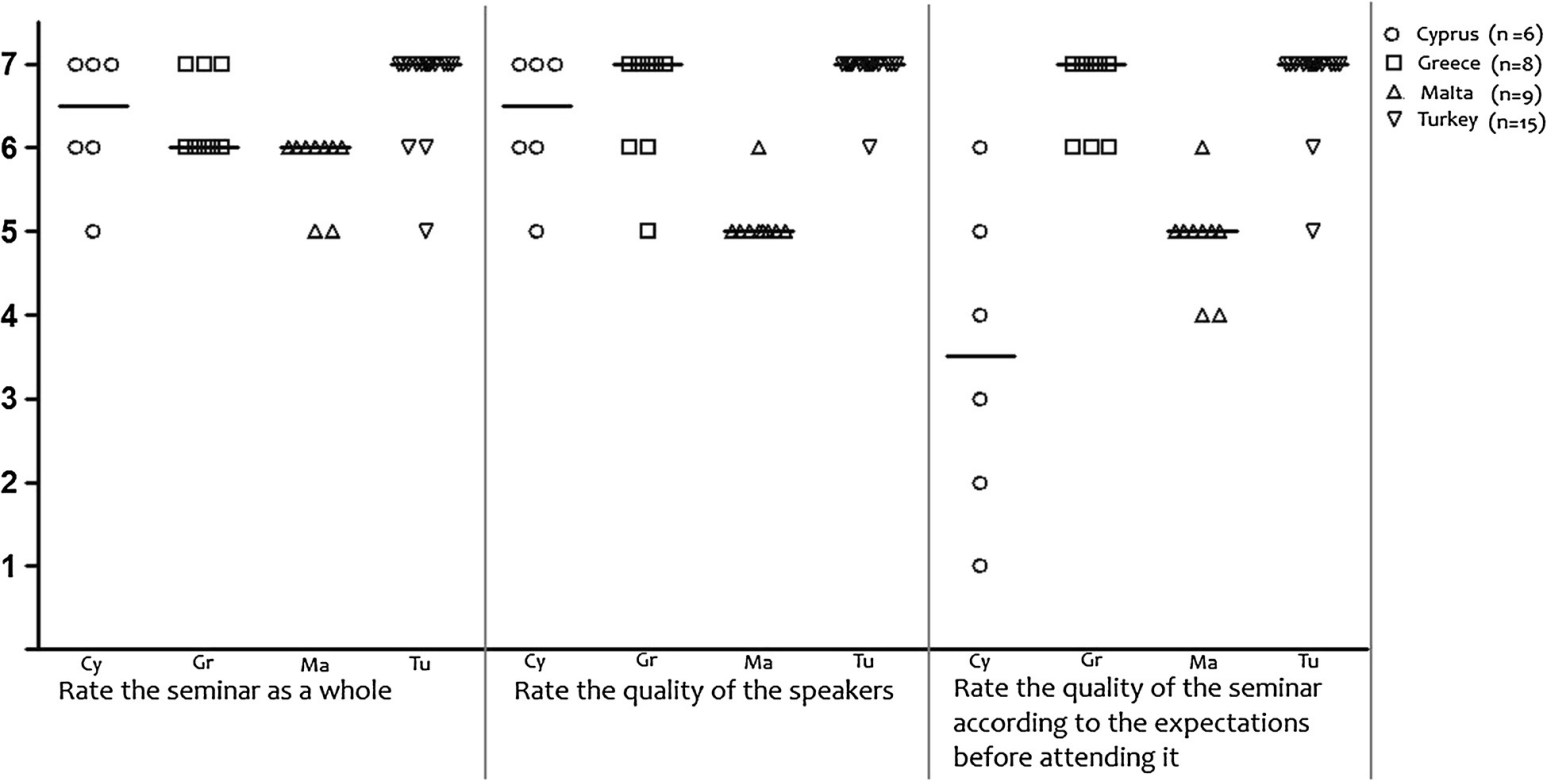
Acceptance related questions (training assessment questionnaire).

The organization and content of the training received positive evaluation by all participating countries with median scores being equal or greater than 5 on a 7-point Likert scale (Figure 1). The material of the intervention was positively assessed in Cyprus, France and Turkey (median scores ≥ 6 on a 7-point Likert scale), while it was neutrally assessed in Greece and Malta. The same pattern was reported in the relevant items found in the Training Assessment Questionnaire. The seminar, in its entirety, received high assessment scores (≥ 6 on a 7-point Likert scale), with a very good overall quality of speakers (≥ 5 on a 7-point Likert scale), and it was considered to be of fairly good quality according to the expectations physicians had prior to participation (Figure 2). Together, these results indicate a high degree of acceptance by all participating countries especially those in the East Mediterranean region where the proportion of completed questionnaires was higher.

### Practicality

**Figure 3:**
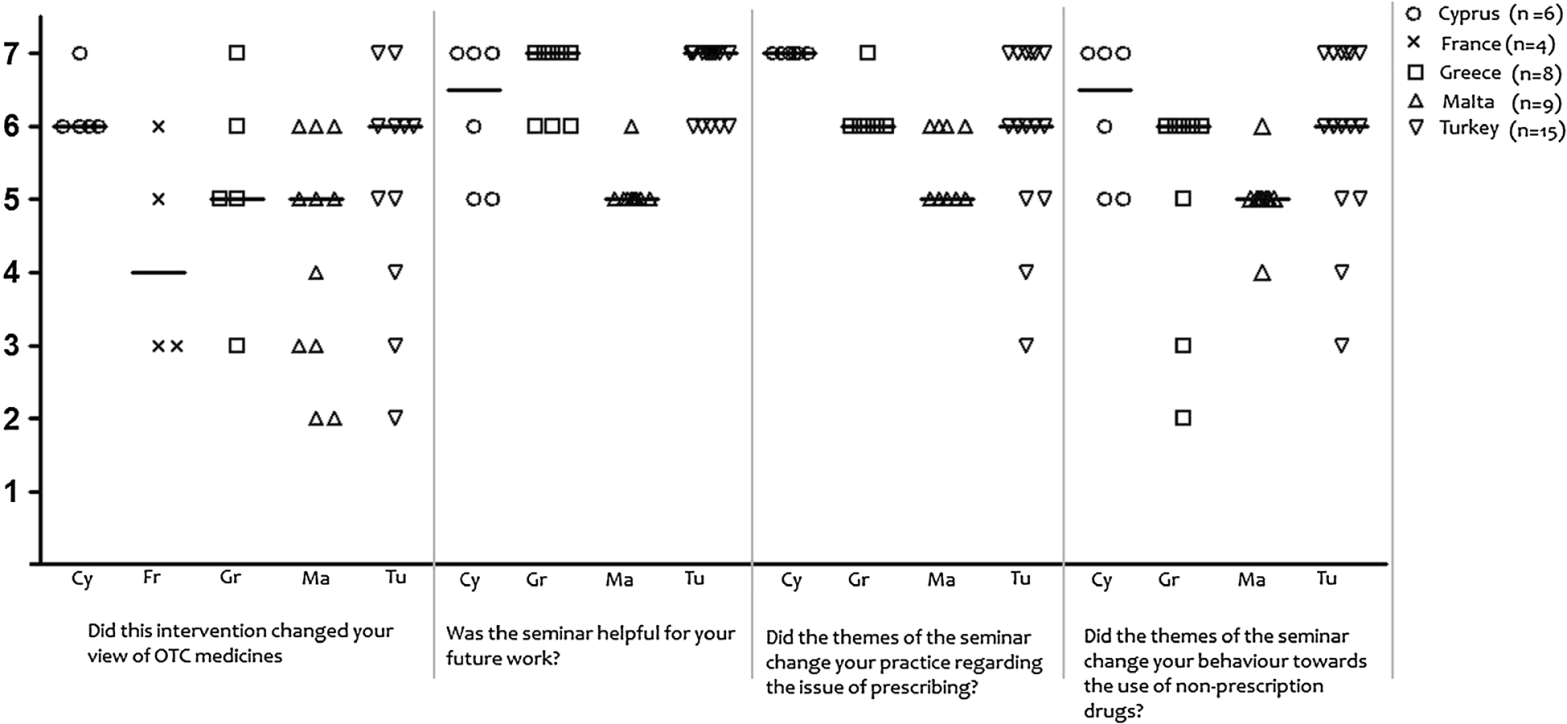
Practicality related questions (complementary questionnaire on OTC medicines & training assessment questionnaire).

GPs from the East Mediterranean countries (Cyprus, Greece, Malta and Turkey) noted that the intervention changed their view on OTC medicines (median scores ≥ 5 on a 7-point Likert scale) while in France that question was neutrally assessed. Based on responses to selected items of the Training Assessment Questionnaire, physicians in all participating countries perceived the seminar as useful for their future practice and considered that its themes could potentially influence their prescribing practices and their views on OTC medicines’ consumption. Higher scores in the above statements were observed in Cyprus, Turkey and Greece compared with Malta (Figure 3).

### Other measures

**Figure 4:**
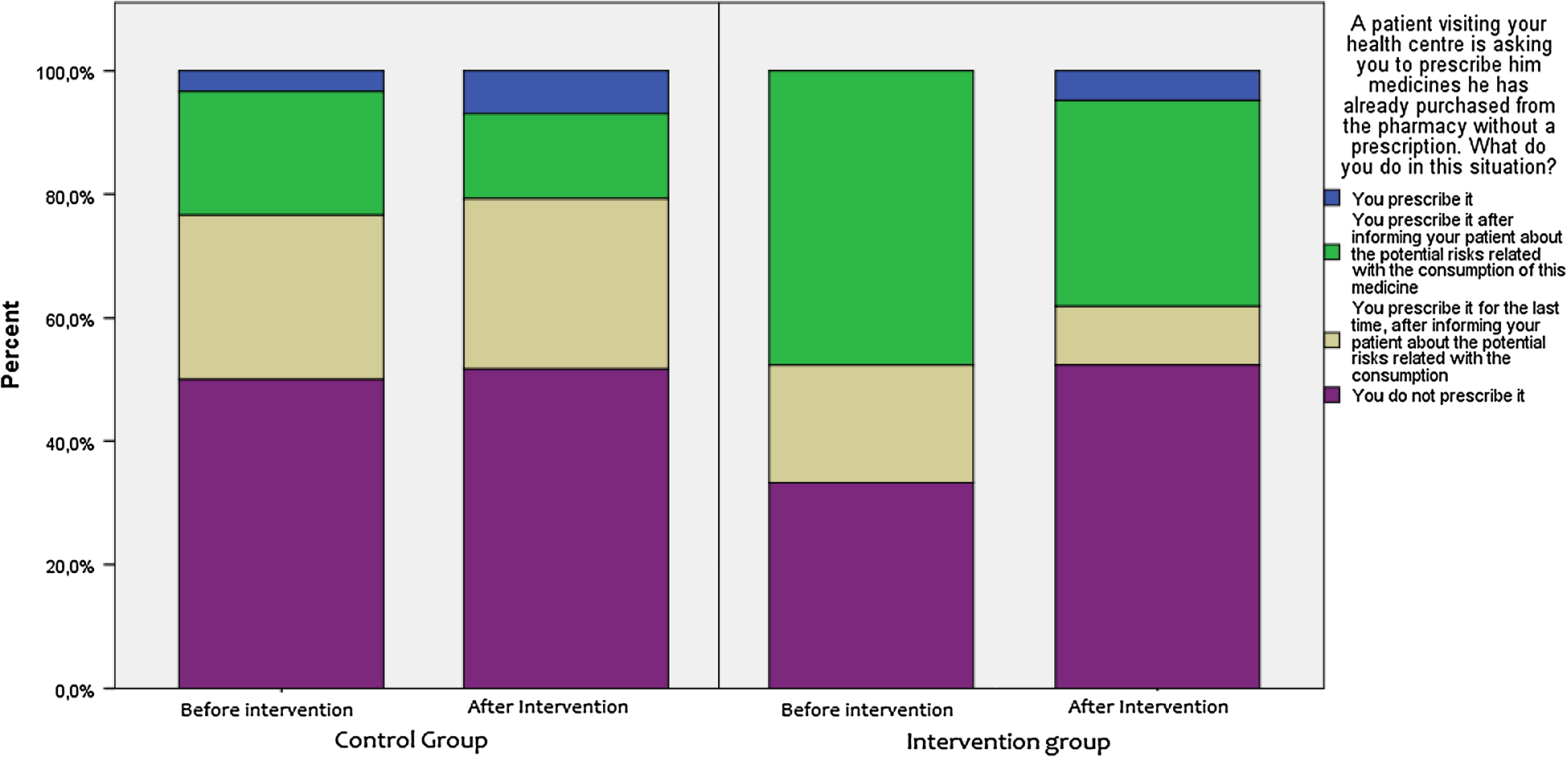
Complementary questionnaire on OTC medicines 1.

**Figure 5:**
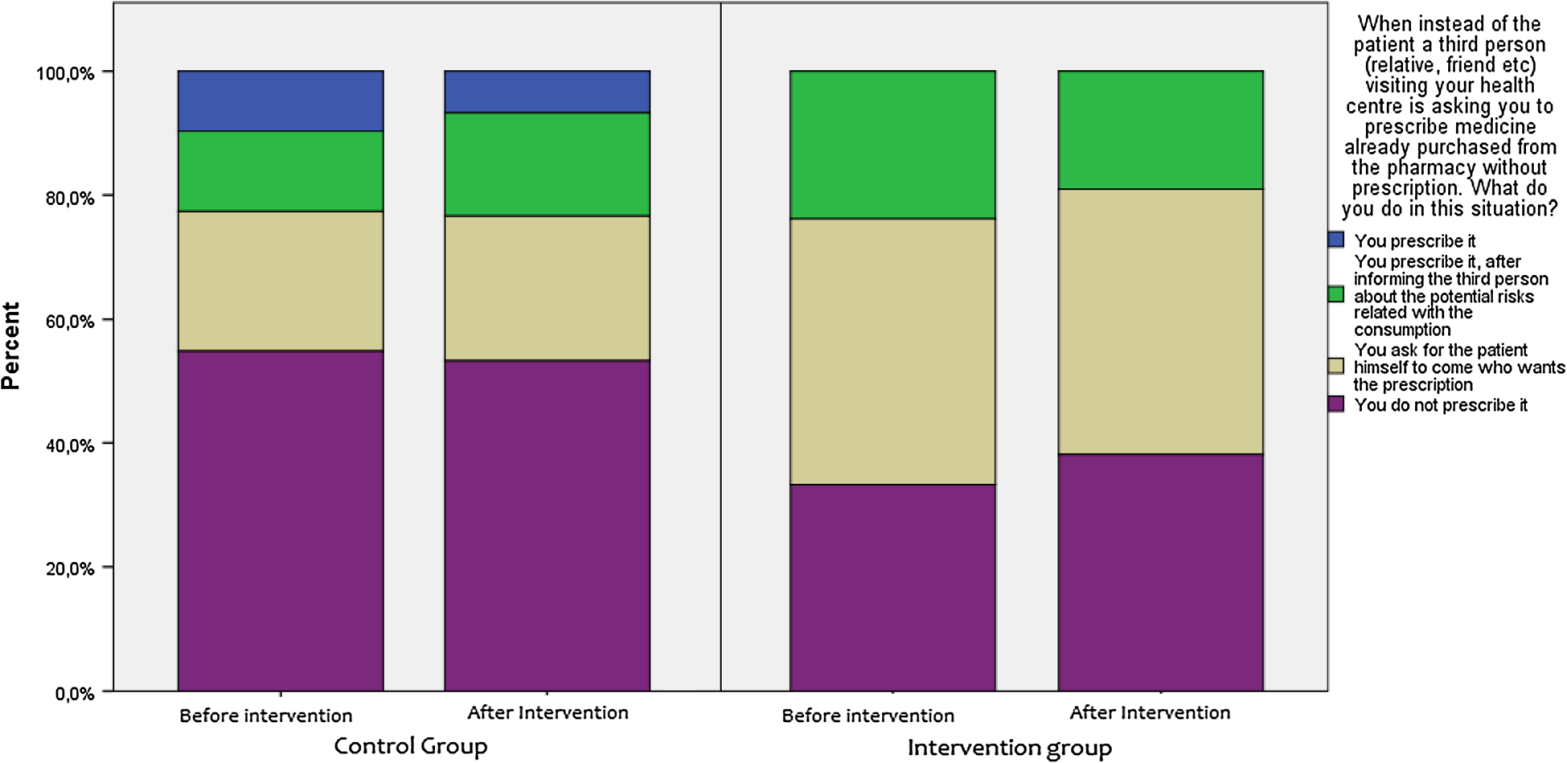
Complementary questionnaire on OTC medicines 2.

**Figure 6:**
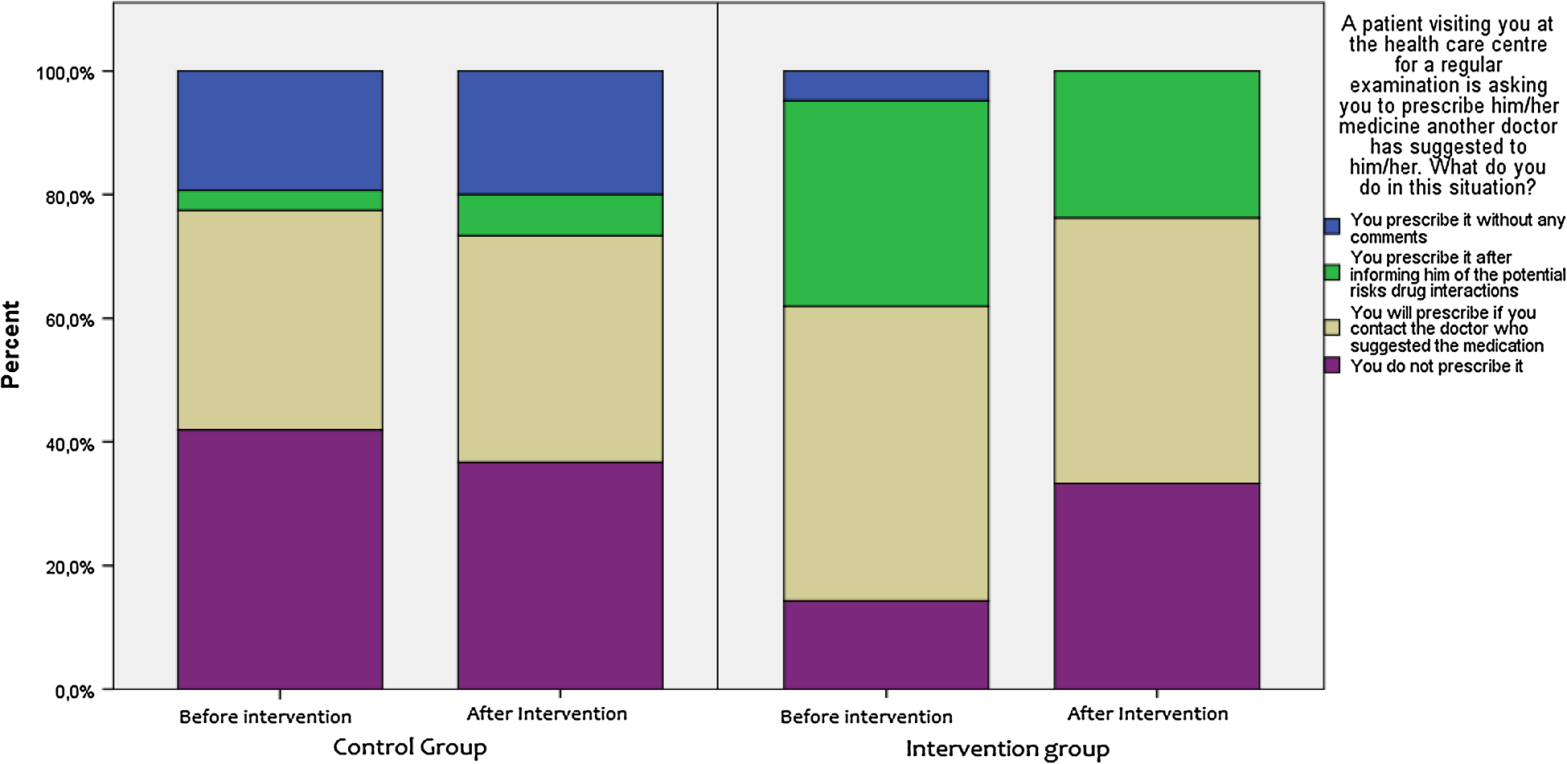
Complementary questionnaire on OTC medicines 3.

**Figure 7:**
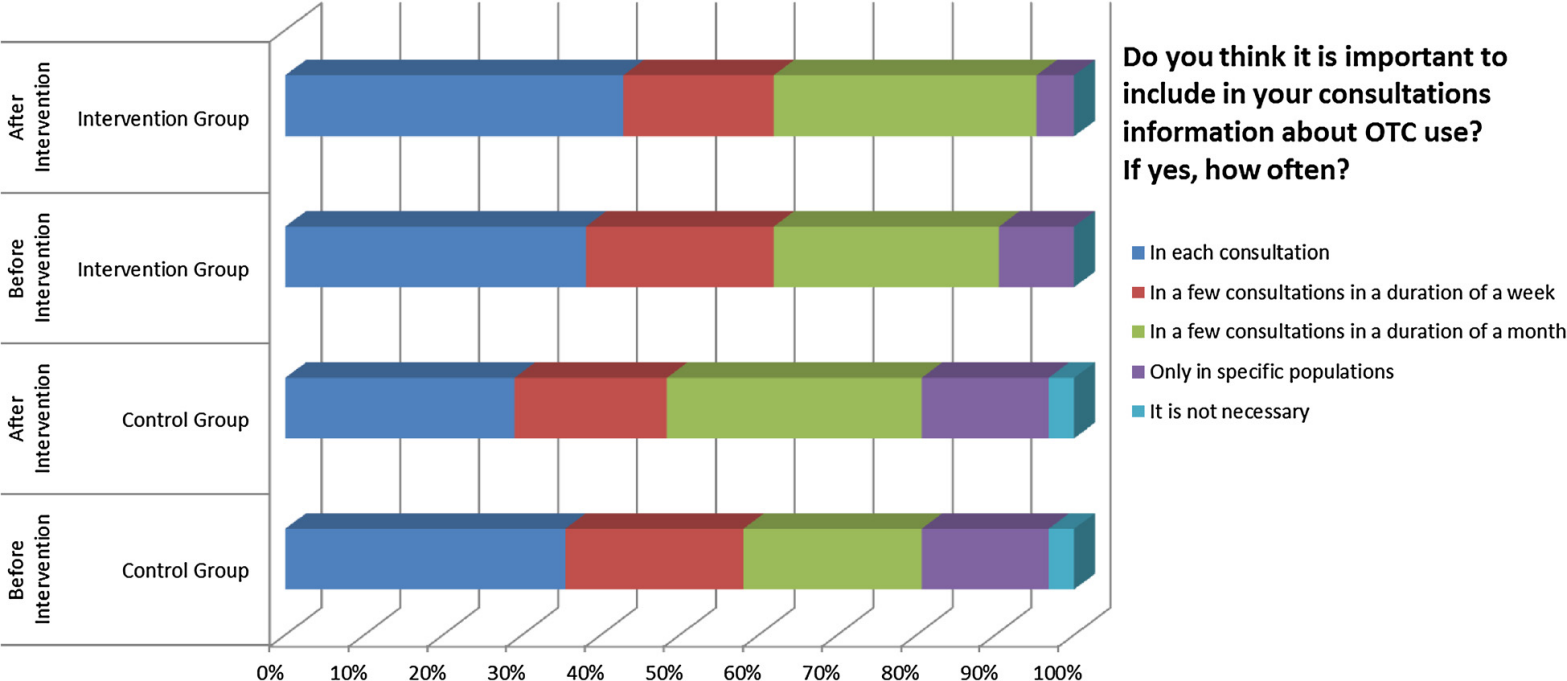
Complementary questionnaire on OTC medicines 4.

Results from the Complementary Questionnaire in OTC medicines also indicated a favourable change towards a more rational prescribing in intervention groups. GPs were asked how they would respond in a situation where a visiting patient or third person (friend/relative of the patient) asked them to prescribe medicines already bought from the pharmacy. The rate of non-compliance with such behaviour was found to be higher after the intervention. A similar pattern was observed when GPs were asked how they would respond in a situation of a regular-visiting patient asking for a medicines’ prescription for medicines suggested by another physician. Post-intervention replies included rational medicine-provision patterns such as contact with the other physician prior to prescription at a higher extent compared to baseline responses (Figures 4, 5 and 6). To explore physician behaviour regarding appropriate use of OTC medicines, GPs were asked whether it is important to include information on OTC medicine use in their consultation and, if so, how often. After the intervention, the frequency of those who responded that they should include information on OTC use in each consultation was increased compared to baseline in the intervention group, while it was reduced in control group. Furthermore, the frequency of those who responded that they should include information on OTC use only in certain cases was reduced in the intervention group contrary to the control group where it remained unchanged. Results are depicted in Figure 7.

## Discussion

### Main findings

The main finding of this feasibility study suggests that an intervention designed on the basis of TPB aimed at modifying GPs’ behaviour towards prescribing or recommending OTC medicines was considered to be well-accepted and practical according to the evaluation by participating physicians. A higher degree of acceptance and practicality was observed in settings from the East Mediterranean region where the problem of irrational use of OTC medicines seems to be more significant. Although the feasibility study indicated a limited efficacy regarding physicians’ prescribing patterns as measured by the TPB, the data of this study show a favourable intention towards irrational prescription. Additionally, the study adds evidence confirming that translating theory into practice is feasible.

To our knowledge, such an intervention has not been previously implemented in this geographic area, where rational prescribing and use of OTC medicines and pharmaceutical costs are key issues for the current health care and health policy agenda [51-53].

Evidence from this study indicated that the intervention that was carried out could play a significant role towards the improvement of the previously described current conditions. GPs’ intentions, as approached by the TPB, were reduced post-intervention towards less favourable attitudes regarding medicine provision. This pattern was backed up by a reduction in the number of medicines that GPs provided to their patients after the intervention, as observed in Malta and in Turkey. In parallel with the above, a change towards a more rational behaviour in situations regarding prescription to third persons or medicines already bought from pharmacies was observed. It is evident, that the combination of the above findings suggests a well-targeted approach concerning GPs behaviour. On the other hand, this intervention was not directly targeted towards the behaviour modification of pharmacists or patients. It should be stressed that the issue of polypharmacy is not solely dependent on the behaviour of GPs. A schematic representation of these three groups would be a triangle, with GPs on one side, pharmacists and patients on the other two sides. Behaviours and interactions between all sides ought to be approached by a larger scale intervention with multiple targets in order to assess whether such designs could be effective enough to promote a better management of the recorded situation.

### Strengths and limitations

This study attempted to identify the potential impact of an educational intervention on GPs’ prescribing practices. Such interventions have been widely recommended in the international literature [30]. Among the strengths of this intervention is the fact that it was based on a clearly specified theoretical framework, the TPB model, and there is some evidence demonstrating that the concepts of this model are related to behavioural change within the context of prescribing [53]. Additionally, this study was implemented in Mediterranean countries of the Southern European region were such interventions are lacking. The intervention was focused solely on GPs, thus the lack of involvement of pharmacists and/or patients/clients in the intervention represents a limitation. We involved purposively only GPs without inviting patients and pharmacists as the involvement could artificially enhance the effectiveness of the pilot study. Another potential limitation of this study is that its feasibility was tested in selected districts of each participating country where a convenience sampling led to different GP demographics; all of these limitations introduce a degree of bias and may limit the generalisability of the results of the study, and also limit the external validity of the study. A convenience sampling was performed for GP practice selection in participating countries and, thus, our findings may be less amenable to drawing conclusions for the GPs in the various countries. Another limitation of the study is the lack of response rates per country, so while this intervention was found to be well-accepted by participating GPs, one cannot safely infer regarding its acceptance on a larger scale. In addition, it should be noted that the French GP uptake in this study was extremely low. This introduces a high risk in making safe inferences drawn from this particular group or comparisons to other settings, thus, the results of the pilot study at this point should be interpreted with some caution. In addition, the differences on sociodemographic data among the participating GPs may have an impact on the interpretation of the data on effectiveness. Finally, the tools that were used for the evaluation of the intervention were theoretically sound, but not tested in terms of their reliability and validity in the population of interest.

In addition to the above issues, there are concerns regarding existing differences with regards to cultural and organisational heterogeneity, and the dispensing policy within the various primary care setting may have an impact on the interpretation of the study results. Although the background data to the project support that Greece, Malta, Cyprus and Turkey are very similar in the provision of primary care services, it is noted that variations from country to country in terms of the GPs practices, culture and doctor-patient relationship among the participating settings exist and they have been recorded in general terms. We did not adjust the existing differences in the pilot intervention, however; this was out of the scope of the current pilot feasibility study which aimed to assess effectiveness, practicality and acceptance in the local settings. We wish to underline that this issue should be taken into consideration in future RCT studies.

This feasibility study attempted to address the pragmatic conditions existing in each setting. Although certain similarities exist among the different country settings, there are clear differences in the organization of primary care services, in the prevailing health culture of the patients as well as in the demographic characteristics. Certainly, as previously mentioned, this restricts the generalisability of the study findings, and we would draw attention to the fact that these should be interpreted carefully within each unique political and cultural setting*.*

### Impact of the study

This study is particularly timely, as certain European countries are currently facing a financial crisis, while at the same time physicians and pharmacists seem to provide medicines to a large number of patients often as a result of social pressure. This feasibility study, despite its limitations, could provide valuable insights for a large-scale study. Qualitative studies and the analysis of empirical data may prove valuable in highlighting areas of research, which should be taken into consideration when designing such trials. This intervention study also highlighted the GPs’ high expectation for guidance and training and this could be a key issue in health care reforms currently discussed and implemented in Southern European countries. Most importantly, the current intervention was tested in various settings and a proposed intervention frame has been evaluated as feasible, well-accepted and practical in the busy health care environment. The study further provides an operationalized structure to define and evaluate interventions targeting similar behaviours in health professions and other disciplines. It introduces common evaluation standards and tools translated in multiple European languages, appropriate for measuring the effectiveness of current interventions and their applicability in other settings. Researchers now have access to an educational intervention tool with relevant methodologies and instruments for a future large-scale implementation, to alter the existing situation at the regional and national levels, allowing for substantial curbing of pharmaceutical expenditures. Furthermore, the current study provides evidence to policy makers on future policy actions targeting physician skills and prescribing behaviours in primary health care. It can additionally provide guidance on how to manage physician behavioural change and how to prevent irrational prescribing of medicines at primary care settings, through borrowing theoretical constructs from behavioural sciences. These constructs could be used in undergraduate, postgraduate and continuous medical education, to improve medical practice. This study is further expected to enable multi-country, multi-stakeholder consultations regarding long-term planning for the provision and consumption of medicines.

## Conclusions

The content, constructs and methods of the designed pilot intervention study highlighted aspects of feasibility and elements of acceptance although certain methodological issues, including the selection of the different groups within the different European cultural and organisational settings, may have an impact on the interpretation of the results and on future transferability. This study also advocates the implementation of well-designed randomized studies in this field and highlights certain essential components for successful implementation of future interventions and research studies. The results of the current study may provide sufficient information to GPs and health policy makers to promote large-scale research, which is an issue of importance in certain European countries and especially those that have been affected by the financial crisis.

### Ethical approval

Approval has been obtained from the National Health Care Services in Cyprus, France, Greece, Malta and Turkey, within the seventh framework programme of the OTC SOCIOMED project (Grant agreement number 223654).

## Abbreviations

OTC: Over the counter; TPB: Theory of planned behaviour; GP: General practitioner; FP7: Seventh framework programme; PHC: Primary health care; GI: Generalized intention towards medicine provision; IPS1: Intention performance statement 1; IPS1: Intention performance statement 2.

## Competing interests

The authors declare that they have no competing interests.

## Authors’ contributions

CL was the coordinator of the FP7 European project and conceived the idea of this study. CL and GB prepared the first draft, SS, IGT and EP contributed at a next stage and AK, VT, MP, AT, JM, AS, AB, TF, AF, LM, DA, YU, JV, AA, TE and BM all contributed to the study design and the final manuscript. CL, MP, AB and EP contributed to the revision of the manuscript. All authors reviewed and approved this manuscript before its submission.

## Pre-publication history

The pre-publication history for this paper can be accessed here:

http://www.biomedcentral.com/1471-2296/15/34/prepub
